# Supplementary material for: The Interstitial Gland as a Source of Pro- or Anti-Senescent Cells during Chinchilla Rabbit Ovarian Aging
Source: Int J Mol Sci. 2024 Sep 13;25(18):9906. doi: 10.3390/ijms25189906 (PMC11432340; doi:10.3390/ijms25189906)
Supplement: Supplementary file 1 [file ijms-25-09906-s001.zip › ijms-3121864-supplementary.pdf]

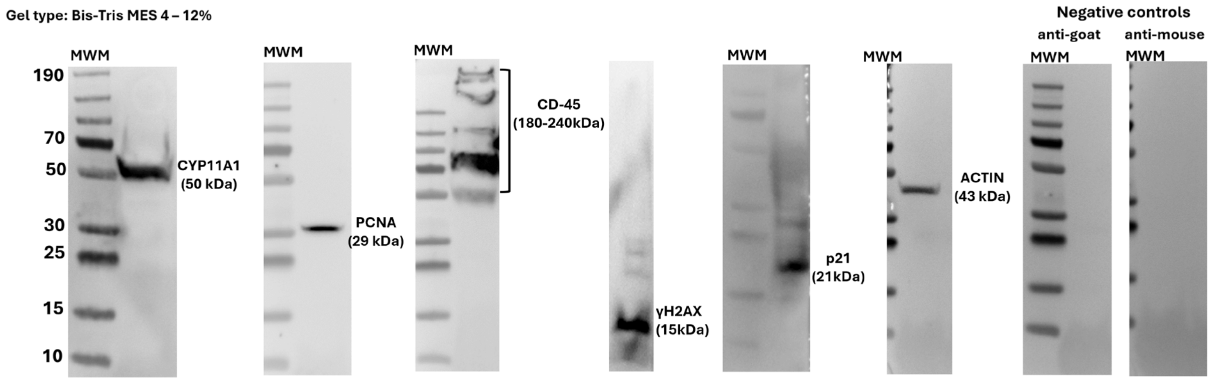

Supplementary Figure S1: Validation of the primary antibodies used in the immunohistochemical study on the ovary of a rabbit aged 30 months old. The protein molecular weight marker (MWM) corresponds to PageRuler Plus Prestained Protein Ladder (Thermoscientific). The negative control was incubated with secondary antibodies overnight, without primary antibodies.
